# Transcriptomic analysis and experiments revealed that remimazolam promotes proliferation and G1/S transition in HCT8 cells

**DOI:** 10.3389/fonc.2024.1345656

**Published:** 2024-04-25

**Authors:** Runjia Wang, Shuai Li, Han Hu, Qi Hou, Huaqing Chu, Yu Hou, Cheng Ni, Yuliang Ran, Hui Zheng

**Affiliations:** ^1^ Department of Anesthesiology, National Cancer Center/National Clinical Research Center for Cancer/Cancer Hospital, Chinese Academy of Medical Sciences and Peking Union Medical College, Beijing, China; ^2^ Department of Anesthesiology, Shanghai Pulmonary Hospital, Tongji University School of Medicine, Shanghai, China; ^3^ State Key Laboratory of Molecular Oncology, National Cancer Center/National Clinical Research Center for Cancer/Cancer Hospital, Chinese Academy of Medical Sciences and Peking Union Medical College, Beijing, China

**Keywords:** remimazolam, cell cycle, proliferation, HCT8 cells, bio-informatics

## Abstract

**Background:**

Remimazolam is a new ultrashort-acting benzodiazepine for sedation and anesthesia. The effects of remimazolam and the mechanism by which it functions in cancer cells have not been determined. This research aimed to explore the mechanism of remimazolam action in colon cancer treatment, using bioinformatics analysis and *in vitro* experiments.

**Methods:**

Cell cycle progression, colony formation, self-renewal capacity, and apoptosis detection were performed in HCT8 cells treated with or without remimazolam. Transcriptome sequencing, Gene Ontology, Kyoto Encyclopedia of Genes and Genome, Protein–Protein Interaction, Gene Set Enrichment Analysis, Western blotting, and qPCR were performed to investigate the mechanism of action of remimazolam in HCT8 colon cancer cells.

**Results:**

Remimazolam promoted proliferation and cell-cycle progression of HCT8 cells. After remimazolam treatment, a total of 1,096 differentially expressed genes (DEGs) were identified: 673 genes were downregulated, and 423 genes were upregulated. The DEGs were enriched mainly in “DNA replication“, “cell cycle”, and “G1/S transition” related pathways. There were 15 DEGs verified by qPCR, and representative biomarkers were detected by Western Bloting. The remimazolam-mediated promotion of cell proliferation and cell cycle was reversed by G1T28, a CDK4/6 inhibitor.

**Conclusion:**

Remimazolam promoted cell-cycle progression and proliferation in HCT8 colon cancer cells, indicating that the long-term use of remimazolam has potential adverse effects in the anesthesia of patients with colon cancer.

## 1 Introduction

Colon tumors are the second most prevalent malignant neoplasm (1), and the second most deadly cancer worldwide (2), with higher rates of metastasis or recurrence at advanced stages than at earlier stages (3). The 5-year survival rate of colon cancer patients is low because of cancer chemotherapy resistance, aggressiveness, metastasis, and relapse. This leads to patients’ demand for surgery and colonoscopy under anesthesia and analgesia during cancer treatment. Remimazolam, a novel ultra-short-acting benzodiazepine site on γ-aminobutyric acid (GABA) (A) brain receptors, is associated with rapid onset and offset of anesthesia and sedation, and minimal hemodynamic instability (4). Remimazolam was approved for conducting in general anesthesia on January 2020 in Japan and November 2021 in China. Remimazolam is safe and effective for bronchoscopy and digestive endoscopy. It offers accurate control of drug effects by titration of the infusion rate and boluses with rapid recovery after administration ceases (5) and provides procedural sedation with higher success rates and recovery profiles than dose midazolam (6). Compared with propofol, the most commonly used intravenous anesthetic, remimazolam, has significant safety advantages in terms of pain, hypotension, and the need for treatment of hypotension, as well as the overall incidence of adverse events, which is significantly lower than that of propofol (7).

As reported, GABA is weakly present in early-stage I samples but is highly abundant in later stage II and III samples in colon adenocarcinoma, indicating that GABA accumulates as cancer progresses, and patients who exhibit low intratumoral GABA content survived longer than those with high GABA (8). GABA type A receptor GABRA2, GABRA3, GABRB2, GABRB3, and GABRG2 may allow differentiation between colon tumor tissues and adjacent normal tissues (9). Moreover, GABA activates the GABA(B) receptor to inhibit GSK-3β activity, leading to enhanced β-catenin signaling that stimulates tumor cell proliferation (8). On the contrary, other researchers reported that the activated GABA(B) receptor induces colorectal cancer cell apoptosis through the inhibition of cAMP-dependent signaling pathways and cellular inhibitor of apoptosis protein 2 (cIAP2) expression (10). The GABAB receptor inhibits tumor progression and epithelial–mesenchymal transition via regulating the Hippo/YAP1 pathway in colorectal cancer (11). Propofol, an anesthetic site on GABA receptors, promoted colon cancer cell metastasis through the GABAAR–TRIM21–Src mechanism and enhanced adhesion and extension of tumor cells to vascular endothelial cells, a critical step in tumor metastasis (12). Also targeting GABA receptors, the effect of remimazolam on cancer cells, especially colon cancer, is unclear. However, there are currently no research reports on the comprehensive effects of GABA receptor anesthetics on residual tumor cells in colorectal cancer patients, especially after long-term and repeated use. It is significant for us to choose suitable anesthetics for surgery and colonoscopy during the treatment of colorectal cancer.

However, little is known about the role of remimazolam in colon cancer. The goal of this research was to identify differentially expressed genes (DEGs) and determine the underlying mechanism of action of remimazolam via bioinformatics and *in vitro* experiments in HCT8 human colon cancer cells.

## 2 Methods

### 2.1 Cell culture and reagents

The LOVO and HCT8 human colon cancer cell lines were obtained from the Chinese Academy of Medical Sciences. Cell lines were cultured with DMEM culture medium (Livning, China) supplemented with 10% fetal bovine serum (BI, Israel) containing 1% penicillin–streptomycin (Livning, China) at 37°C in a humidified atmosphere with 5% CO_2_. Remimazolam (Hengrui Pharmaceuticals Co., China) is water-soluble, at approximately 200 μg/mL, as previously reported (13). G1T28 (MCE, USA), a CDK4/6 inhibitor also named trilaciclib, was dissolved in DMSO and the culture medium. HCT8 cells were treated with 300 nmol/L G1T28 for 24 h.

### 2.2 Cell proliferation assay

LOVO and HCT8 cells were seeded in 96-well plates (1,000 cells/well) and cultured for 24 h. Then, the cells were treated with 200 μg/mL remimazolam for 5 days. Cell Counting Kit-8 (CCK8, Dojindo Chemistry, Japan) reagent was added to every well, and the cells needed to be further cultured for 2 h. The optical density at 450 nm was detected by a microplate reader (Bio-Rad, USA). By measuring the optical density of each well, the cell viability curve was determined, and the results were average of three wells.

### 2.3 RNA isolation and library preparation

Total RNA was extracted from HCT8 cells treated with or without remimazolam with TRIzol (Invitrogen, USA) according to the protocol. RNA quantification and purity were evaluated by a NanoDrop 2000 spectrophotometer (Thermo Scientific, USA). RNA integrity was assessed with Agilent 2100 Bioanalyzer (Agilent Technologies, USA). The libraries were constructed using the VAHTS Universal V6 RNA-seq Library Prep Kit. Transcriptome sequencing and analysis were performed by OE Biotech Co., Ltd. (Shanghai, China).

### 2.4 RNA sequencing and differentially expressed gene analysis

The RNA-seq libraries were subsequently sequenced on an Illumina NovaSeq 6000 platform. There were 150-bp paired-end reads generated. First, fastp was used to process the raw readings in the fastq group. Low-quality readings were removed for clean readings. HISAT2 was used to map clean reads to the reference genome. The FPKM of each gene was calculated. Reads of each gene were obtained via HTSeq-count. Principal component analysis [R (v 4.2.1)] was used to evaluate the biological duplication of the samples.

We used DESeq2 to perform differential expression gene analysis. Q value <0.05 and fold change >2 or fold change <0.5 were set as the thresholds for vital DEGs. R (v 4.2.1) was used for hierarchical cluster analysis of DEGs to evaluate the expression patterns of genes in different populations and samples. GO and Kyoto Encyclopedia of Genes and Genomes (KEGG) pathway enrichment analyses of DEGs were performed based on the hypergeometric distribution, and significant enrichment items were screened by R (v 4.2.1). R (v4.2.1) was used to construct the column, bubbles, and chord diagrams illustrating the important enrichment terms.

Gene set enrichment analysis (GSEA) was also conducted using GSEA software. The analysis was performed on a predefined gene set, and these genes were ranked according to the level of fluxionary expression in the two sample types. Finally, we tested whether the predefined gene set was enriched at the bottom or top of the ranking list.

### 2.5 Construction of protein–protein interaction network

The STRING database (https://string-db.org/) contains more than 52 million proteins whose interactions were analyzed. The protein–protein interaction (PPI) network of the top 30 targets was created using Cytoscape tools and STRING, to help decipher their relationships. The circle graph of the top 30 PPI relationships was drawn by ordering the combined interaction scores from highest to lowest. The greater the association between genes, the greater the number of gene points.

### 2.6 Colony formation assay

There were 1,000 HCT8 and LOVO cells respectively treated with or without remimazolam which were cultured in six-well plates. The cells were fixed with 4% paraformaldehyde (Livning, China) for 30 min after 10 days of culture. After washing with phosphate-buffered saline (PBS), the colonies were stained with 5% crystal violet (Livning, China) for 15 min and assessed by two investigators’ visual. Each experiment was performed in triplicate with two biological replicates per group.

### 2.7 Western blot and antibodies

Protein from each sample (20 µg) was separated by 10% SDS-PAGE and electro-transferred to a PVDF membrane (Millipore, USA) for immunoblot analysis. Membranes were blocked and incubated with the corresponding primary antibody at 4°C overnight. Then, the cells were incubated with the corresponding secondary antibody. The membranes were washed three times with TBST. Antibody binding was detected by enhanced chemiluminescence reagent kit (Livning, China) and photographed with ImageQuant LAS 4000 CCD camera (GE Healthcare, USA). The catalog numbers of the antibodies are provided in **Supplementary Document**.

### 2.8 Cell apoptosis detection

HCT8 cells were cultured in six-well plates with or without remimazolam treatment. Then, cells in each well were collected and washed with cold PBS. The detached cells were suspended in Annexin V binding buffer and then stained with Annexin V-FITC/PI (Biosharp, China). The cells were incubated for 10 min at room temperature in the dark after gently mixing. Apoptosis was detected via FACS (Attune NxT, Thermo Fisher, USA).

### 2.9 5-Ethynyl-2′-deoxyuridine/PI staining and flow cytometry analyses

5-Ethynyl-2′-deoxyuridine (EdU) is incorporated into genomic DNA during DNA synthesis in the S phase of the cell cycle. EdU staining of HCT8 cells was conducted using the cell-light EdU Apollo643 *in vitro* kit (C10310-2, Ribobio, China), fixatives, Click-iT™ saponin permeabilization solution, and wash buffers of Click-iT™ EdU Alexa Fluor™ 488 Kit (Thermo Fisher, USA). EdU solution (reagent A) was diluted 1,000:1 to produce a 50-μM EdU solution. Then, in each well, the culture medium was replaced with EdU (1 mL) medium and incubated for 2 h. HCT8 cells with or without remimazolam treatment were collected into a flow tube and centrifuged, after which the supernatant was discarded. Cells were resuspended in 1 mL PBS and washed. After adding 1 mL of 4% paraformaldehyde (Livning, China) for 30 min, each tube was centrifuged and the supernatant was discarded. The solution was neutralized by adding 2 to 3 mL of 2 mg/mL glycine for 5 min, after which the tube was centrifuged, washed once in 2 mL PBS, and centrifuged, after which the supernatant was collected. Each tube was incubated with 1 mL of 0.5% Triton X-100 at room temperature for 15 min, and the supernatant was centrifuged and then washed with 2 mL PBS. After adding 500 μL of 1× Apollo® staining solution to each tube, the cells were fully resuspended, incubated without light at room temperature for 15 min, and centrifuged and the staining solution was discarded. Each tube was washed two times with 3 mL of 0.5% Triton X-100 at room temperature, centrifuged to discard the supernatant, and resuspended in 500 μL PBS. After washing with PBS, cells were stained in 1 mL PI staining solution (MCE, USA) for 30 min in the dark. Cell-cycle distribution was analyzed via flow cytometry (ID7000, SONY, Japan), and the data were analyzed via FlowJo v10.8.1.

### 2.10 qPCR assay

According to the TRIzol (Invitrogen, USA) reagent protocol, total cellular RNA was isolated from HCT8 cells treated with or without remimazolam. The cDNA synthesis was conducted using the Transcriptor First Strand cDNA Synthesis kit (Servicebio, China). Using the SYBR® Green PCR Master Mix (Servicebio, China), RNA expression levels were assessed by quantitative RT-PCR. The real-time PCR thermocycling conditions were 95°C for 3 min, followed by 40 cycles (95°C) for 20 s, primer-dependent annealing temperatures on each primer (57°C–60°C) for 20 s, and extension (72°C) for 30 s. Data were presented as the relative expression of each target gene and were calculated by the comparative 2^−ΔΔCT^ method (14). The primers of genes are shown in the **Supplementary Document**.

### 2.11 Self−renewal assay

We used spheroid-formation experiments to explore the self-renewal ability of HCT8 cells. The cells were seeded in 24-well ultra-low attachment plates (Corning, USA) after treatment with or without remimazolam. There were 500 cells per well cultured in serum-free DMEM/F12 medium supplemented with 0.8% methylcellulose (Sigma-Aldrich, USA), 20 ng/mL EGF, B27 (1:50), and 20 ng/mL bFGF. The cells were cultured at 37°C in 5% CO_2_ for 14 days. Then, the spheroids were counted under a microscope.

### 2.12 Statistical analysis

All data were expressed as the mean ± standard deviation (SD) of three independent experiments. The difference was statistically significant according to unpaired Student’s t tests, and results were considered significant if P < 0.05. GraphPad Prism 8.0 and SPSS 26.0 were used to perform all analyses.

## 3 Results

### 3.1 Remimazolam promoted proliferation of HCT8 cells

**Figure 1:**
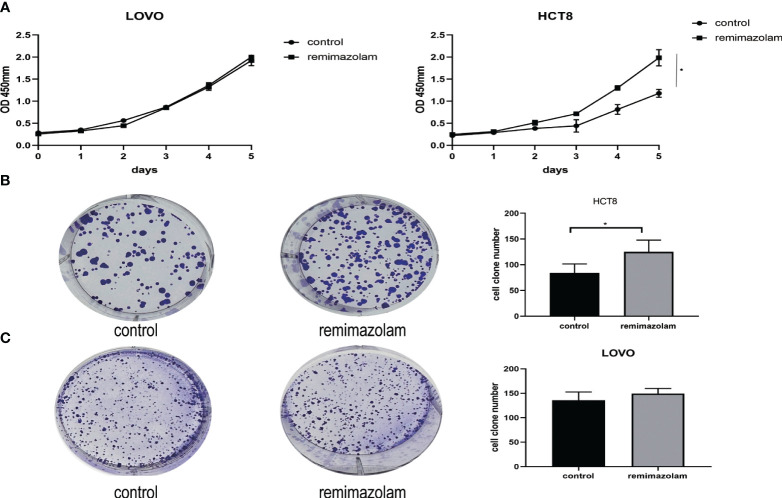
Remimazolam promoted the proliferation of HCT8 cells. **(A)** CCK-8 proliferation curves of the LOVO and HCT8 cell lines. **(B)** Colony formation of HCT8 cells treated with or without remimazolam. **(C)** Colony formation of LOVO cells treated with or without remimazolam. Mean ± SD, *p < 0.05.

LOVO and HCT8 cell lines were treated with 200 µg/mL remimazolam for 5 days as previously reported (13). The results illustrated that remimazolam increased the survival rate of HCT8 cells (**Figure 1A**, p < 0.05) but not LOVO. Remimazolam also increased colony formation number of HCT8 cells (**Figure 1B**, p < 0.05) but not LOVO cells (**Figure 1C**). In summary, these findings indicate that remimazolam promotes proliferation of HCT8 cells. As shown in **Figure 1A** that the survival rate of HCT8 changed on day 3, we treated HCT8 cells with 200 µg/mL remimazolam for 3 days for subsequent experiments.

### 3.2 Gene expression and differentially expressed gene analysis

**Figure 2:**
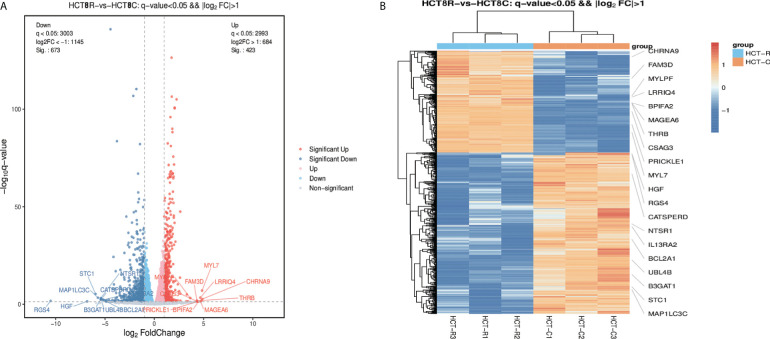
Differentially expressed genes in patients treated with or without remimazolam. **(A)** Differentially expressed genes (DEGs, p < 0.05) in patients treated with or without remimazolam. The red dots represent upregulated genes, and the blue dots represent downregulated genes. **(B)** Heatmaps indicating the expression patterns of DEGs. Red rectangles indicate upregulated genes, and blue rectangles indicate downregulated genes. The deepest color indicates a significant difference.

The DEGs were collected and analyzed. The volcano plot and heatmap were firstly generated. Highlighted red dots indicate genes with significantly upregulated expression (p < 0.05), and highlighted blue dots indicate genes with significantly downregulated expression (p < 0.05). There were 1,096 DEGs in total: 673 genes were downregulated and 423 genes were upregulated following remimazolam treatment (**Figure 2A**). Heatmap showed the expression patterns of DEGs (**Figure 2B**). Red rectangles and blue rectangles indicate upregulated genes and downregulated genes respectively, and more intense colors indicate significant differences.

### 3.3 GO entries, KEGG pathway enrichment, “PPI” network and “cell cycle”, and “DNA replication” were enriched in GSEA

**Figure 3:**
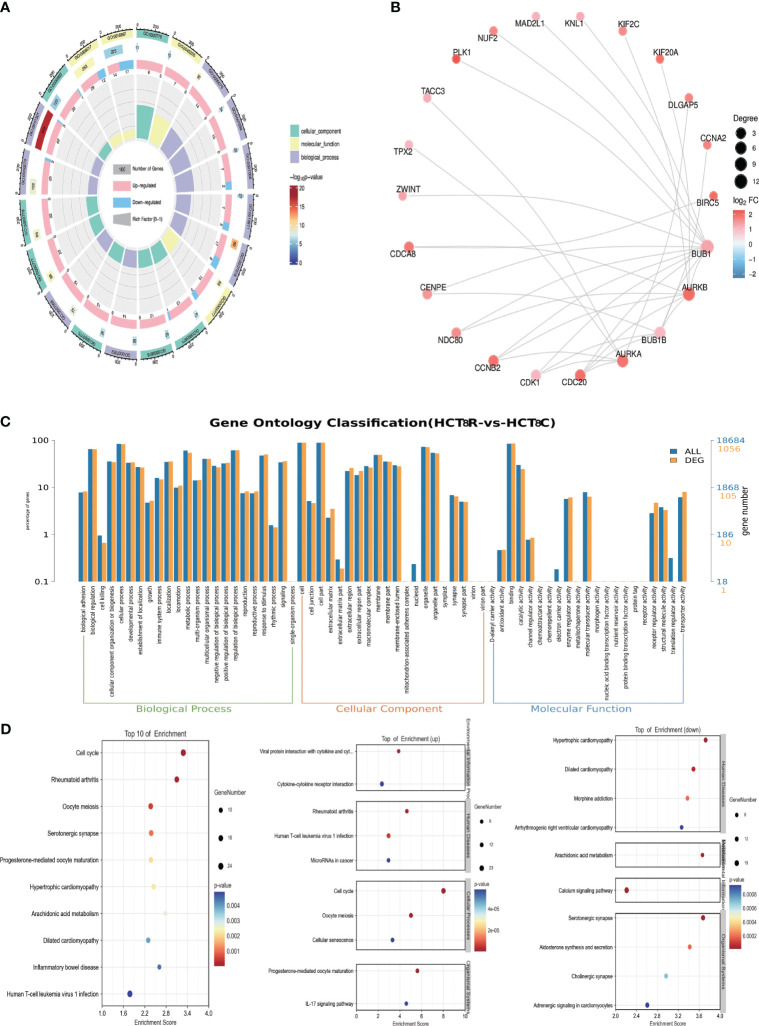
GO, KEGG, and PPI analyses **(A)** Circos plot of the top 20 GO terms. **(B)** Top 30 PPI networks. **(C)** Comparison of the distributions of DEGs and all genes at GO level 2. **(D)** Bubble plot of the top 10 KEGG pathways.

We next performed GO and KEGG pathway enrichment analyses. The 20 categories with the lowest q-value or p-value are displayed in **Figure 3A**. The PPI network with top 30 nodes is shown in **Figure 3B**. Red represents upregulated DEGs, whereas blue represents downregulated DEGs. The distribution of DEGs and all genes at GO level 2 is shown in **Figure 3C**, illustrating the number of genes identified in the biological process category. KEGG pathway analysis was subsequently performed to identify the enriched signaling pathways. **Figure 3D** shows the top 10 enriched KEGG pathways and the up or down KEGG pathways. The cell cycle was shown to be associated with the top 10 pathways and upregulated pathways, demonstrating that the identified DEGs were indeed related to the cell cycle.

**Figure 4:**
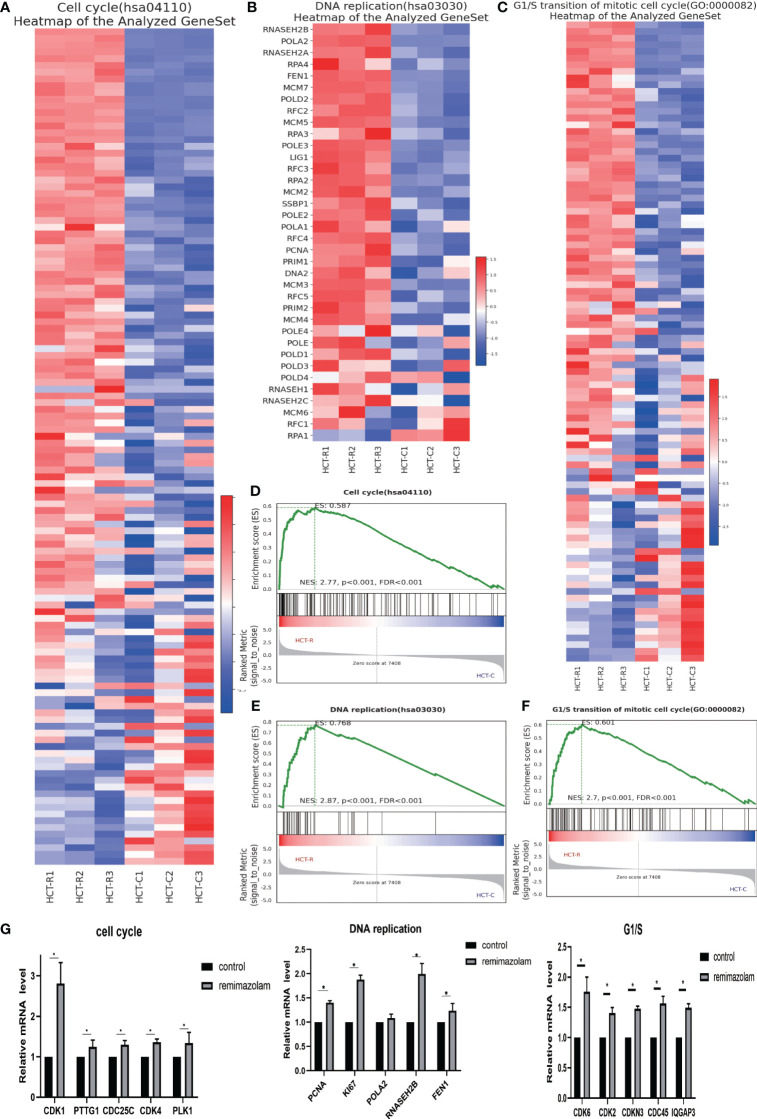
GSEA and qPCR. **(A)** GSEA heatmap of the cell cycle. **(B)** GSEA heatmap of DNA replication. **(C)** GSEA heatmap of G1/S transition. **(D–F)** GSEA map of the cell cycle, G1/S transition, and DNA replication. **(G)** qPCR verification of the genes. Mean ± SD, *p < 0.05.

To explore the functions of the identified DEGs and the pathways in which they function, we conducted GSEA. The GSEA indicated that the DEGs were enriched in “cell cycle”, “G1/S transition”, and “DNA replication” related pathways (**Figures 4D–F**), and most of the DEGs in these three pathways were upregulated (**Figures 4A–C**). DNA replication is a vital part of proliferation. The expression levels of genes whose expression significantly differed after remimazolam treatment, as determined by RNA sequencing, were estimated by qPCR. As expected, the expression level of cell-cycle genes (CDK1, PTTG1, CDC25C, CDK4, PLK1), G1/S transition genes (CDK6, CDK2, CDKN3, CDC45, IQGAP3), and DNA replication genes (PCNA, Ki67, RNASEH2B, FEN1) were significantly (p < 0.05) increased after treatment with remimazolam compared with those in untreated control cells (**Figure 4G**). These *in vitro* results, combined with the RNA sequencing results, indicate that remimazolam promotes the proliferation and cell-cycle progression of HCT8 cells.

### 3.4 Remimazolam promoted cell-cycle progression and growth *in vitro*, and the remimazolam-mediated promotion of cell proliferation and cell-cycle progression was rescued by G1T28 treatment

**Figure 5:**
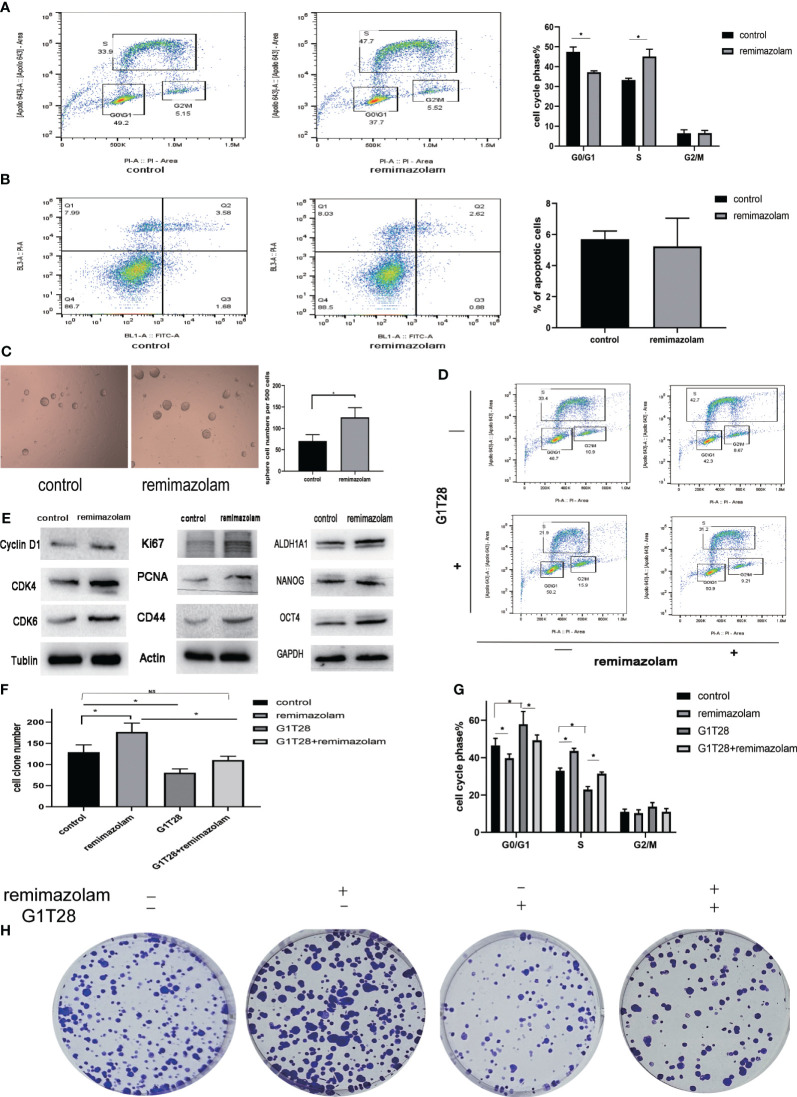
Remimazolam promoted cell-cycle progression, cell proliferation, and stemness, and G1T28 reversed cell-cycle progression and cell proliferation after remimazolam treatment. **(A)** Two-dimensional flow cytometry analysis of EdU incorporation and PI incorporation in cells treated with or without remimazolam. **(B)** Flow cytometry analysis of apoptosis in cells treated with or without remimazolam. **(C)** Sphere formation in cells treated with or without remimazolam. **(D, G)** Two-dimensional flow cytometric analysis of HCT8 cells with or without remimazolam and G1T28 treatment. **(E)** Western blot analysis of biomarkers of cell-cycle progression, proliferation, and stemness with or without remimazolam treatment. **(F, H)** HCT8 cell colony formation with or without treatment with remimazolam and G1T28. Mean ± SD, *p < 0.05.

To monitor cell-cycle progression, two-dimensional flow cytometry analysis of EdU incorporation and PI was used. HCT8 cells were incubated with EdU and PI to label them with both PI and EdU-Apollo 643. G0/G1, S, and G2/M phases were determined by bivariate analysis (15). Dual positive cells positive for both PI and EdU-Apollo643 were considered in the S-phase, and EdU incorporation identifies full-S and early S cohorts (15). After treatment with remimazolam, the proportion of S-phase cells increased and the proportion of G1 phase cells declined (**Figure 5A**, p< 0.05), indicating that cell cycle progression of HCT8 cells during the G1/S phase transition was promoted. Apoptosis was not affected (**Figure 5B**).

Remimazolam induced a significant increase in the number of tumor spheres (**Figure 5C**). We also examined the expression of cell cycle, proliferation, and stemness-related proteins after remimazolam treatment. Western blot analysis indicated significant increases in the expression of Cyclin D1, cyclin-dependent kinase (CDK)4, CDK6, Ki67, PCNA, and CD44 after remimazolam treatment (**Figure 5E**). We also examined markers of stemness, which is related to proliferation and growth (**Figure 5E**). Therefore, remimazolam promoted proliferation, growth, and G1/S transition through the cell cycle in HCT8 cells.

Bioinformatics analyses revealed that remimazolam upregulated CDK1 and CDK4 whereas western blot analysis revealed increases in CDK4 and CDK6; thus, we used G1T28 (trilaciclib), a highly effective and selective reversible inhibitor of CDK4/6, for rescue experiments. G1T28 inhibited the remimazolam-mediated increase in colony formation (**Figures 5F, H**) and proportion of cells in the S phase (**Figures 5D, G**). These results suggest that the remimazolam-mediated promotion of G1/S transition and proliferation can be blocked by an inhibitor of CDK4/6.

## 4 Discussion

This study explored the effects and mechanism of action of remimazolam on colon cancer via bioinformatics analysis and *in vitro* experiments. We found that remimazolam promoted the proliferation and cell-cycle progression of HCT8 cells. By bioinformatics, we found 1,096 DEGs and identified 673 genes whose expression was downregulated and 423 genes whose expression was upregulated upon remimazolam treatment. *In vitro*, we illustrated that remimazolam promoted proliferation and the G1/S transition in HCT8 cells, further revealing that remimazolam significantly regulates the expression of proliferation and cell-cycle components and that G1T28, which is a CDK4/6 inhibitor also named trilaciclib, reversed the promoting effects of remimazolam. In clinic, remimazolam may be used cautiously for long term or more times in patients with colon cancer or that G1T28 may be used in combination with remimazolam.

Colon cancer negatively impacts human life worldwide by reason of its high incidence and mortality rates, which are attributed to a lack of effective drugs for treatment. Anesthesia and surgery are significant components of colon cancer treatment. As a classical benzodiazepine anesthetic, midazolam is widely used. Midazolam induces apoptosis in human cancer cells and inhibits tumor growth *in vivo* (16–18) and has also been shown to suppress cell proliferation and epithelial–mesenchymal transition (EMT) in lung and breast cancer (19). However, a meta-analysis of observational epidemiological studies indicates that using benzodiazepine is associated with increasing risk of cancer (20). The effect of the new ultrashort-acting benzodiazepine anesthetic remimazolam on colon cancer has not been explored before. First, we used CCK-8 and colony formation assays to investigate whether remimazolam affects the proliferation of cancer cells. The phenotype of patients treated with remimazolam differed from that of patients treated with midazolam. To investigate the specific mechanisms through which remimazolam exerts these effects, we performed transcriptome sequencing of HCT8 cells treated with or without remimazolam treatment. In this study, RNA sequencing and differentially expressed gene analysis revealed that the expression of genes related to the cell cycle, G1/S transition, and DNA replication in HCT8 cells were upregulated after remimazolam treatment. We also used combined EdU and PI staining to verify that remimazolam promoted the proliferation and cell-cycle progression of HCT8 cells.

Integrated bioinformatics analysis is a significant approach for identifying main genes and pathways involved in disease pathogenesis (21). Our analysis revealed a total of 1,096 DEGs following remimazolam treatment, of which 673 genes were downregulated and 423 genes were upregulated. After GO and KEGG pathway enrichment, “Cell cycle”, “G1/S transition”, and “DNA replication” related pathways were identified as enriched based on the experimental results. Transcriptome sequencing revealed that cell-cycle-related genes especially G1/S transition genes, CDK1, pituitary tumor transforming gene-1 (PTTG1), Cell Division Cycle 25C (CDC25C), Polo-like Kinase 1(PLK1), CDK4, CDK6, CDK2, CDKN3, CDC45, and IQ Motif Containing GTPase Activating Protein 3 (IQGAP3) were upregulated after remimazolam treatment, and these findings were confirmed by qPCR. We also showed that Cyclin D1, CDK4, and CDK6 protein levels were upregulated after remimazolam treatment. CDK1, CDK2, CDK6, and CDKN3, members of a family of cell-cycle regulatory proteins, are involved in cell-cycle maintenance and are associated with poor overall survival and relapse-free survival (22). CDC25C and CDC45 are closely related to tumor development and tumorigenesis (23, 24). Cyclin CDC25C is included in the specific phosphatase family, which stimulates the cyclin B1/CDK1 complex in cells to enter mitosis, regulating G2/M progression, playing a significant role in checkpoint protein regulation in the presence of DNA damage, and ensuring accurate transmission of DNA to daughter cells during cell division (23). CDC45 plays a significant role in the replicative helicase holoenzyme CDC45-MCM-GINS complex and is highly expressed in human cancer-derived cells (24). CDK4 and CDK6 are vital mediators of cellular transition into the S phase. They are significant for the initiation, growth, and survival of many cancer types (25). CDK4/6 inhibitors are under clinical trials, and palbociclib has received accelerated approval as a treatment for breast cancer (26). PTTG1 is an oncogene in several types of human cancers, an independent poor prognostic factor for patients with colorectal tumor (27). PLK1 is highly expressed in majority of human tumors. PLK1 plays many roles in the cell cycle. It is essential for maintaining genome stability during mitosis and for precisely regulating the cell division, spindle assembly, and DNA damage response (28). IQGAP3 is a critical regulator of mitotic progression and genome stability; thus, it governs proliferation and migration of numerous cancers (29). These results indicate that remimazolam promoted the cell-cycle progression in HCT8 cells, which may exert potential adverse effects on patient survival.

Transcriptome sequencing and qPCR results showed that PCNA, Ki67, RNASEH2B, and Flap endonuclease 1 (FEN1), which are involved in DNA replication, were upregulated after remimazolam treatment. At the protein level, PCNA, Ki67, and CD44 were upregulated after remimazolam treatment. Ki67 and PCNA are representative proliferation-related molecular markers, which are widely used to evaluate the proliferative capacity of cancer cells. CD44 has been widely implicated as a cancer stem-cell marker in many cancers, and high CD44 expression strongly contributes to raised tumorigenic mechanisms, such as cell division, proliferation, and metastasis (30). RNase H2 is composed of a single catalytic subunit (A) with two non-catalytic subunits (B and C), specifically degrading the RNA component of RNA–DNA hybrids. The protein which is encoded by this gene is the non-catalytic B subunit of RNase H2, which plays a role in DNA replication. Increased RNASEH2B expression has also been shown to correlate with metastasis in colorectal tumors (31). FEN1 is a core protein in the base excision repair pathway and participates in Okazaki fragment maturation during DNA replication, which is an effective method for treating cancer as a monotherapy or in combination with other treatments (32). These results indicate that remimazolam promoted DNA replication and proliferation in HCT8 cells. G1T28, also named trilaciclib, is a potent and selective reversible inhibitor of CDK4/6. G1T28 rescued the remimazolam-mediated promotion of cell-cycle progression and colony formation. These findings further indicate that CDK4/6 are targets of remimazolam.

In summary, we identified the potential mechanism by which remimazolam promoted the cell-cycle progression and proliferation of HCT8 cells, which occurred in part by activating representative biomarkers. In this experiment, the duration of remimazolam administration is much longer than that currently in clinic, indicating that the trend of remimazolam on cancer cells is likely to occur and that remimazolam may exert potential adverse effects on patients with colon cancer. Further evaluation of safety of remimazolam for colon cancer patients is needed if longer duration of administration is explored, such as ICU sedation. Minimal residual disease (MRD) of tumor patients is small amounts of cancer cells remaining in the body after cancer treatment, or cancer cells that are unresponsive or resistant to treatment (33). Traditional imaging or experimental methods cannot detect MRD, but they may lead to cancer recurrence. Patients with colorectal cancer need to undergo colonoscopies with anesthesia before and after surgery. More times of use of remimazolam may affect MRD growth, which is detrimental to patient prognosis.

However, this study also has some limitations. First, additional cancer cell lines with different phenotypes should be tested in further investigations, and proliferation should be assessed with additional techniques. Second, our results should be further verified *in vivo*. Third, the exact mechanism by which remimazolam interacts with the receptor of HCT8 was not explored. Because remimazolam has been approved for use in China for only 3 years, we have not yet obtained clinical data related to patient prognosis; thus, additional investigation of subpopulations of patients with cancer should be performed.

## 5 Conclusion

This study revealed that remimazolam promoted the cell-cycle progression and proliferation of HCT8 colon cancer cells by upregulating CDK1, PTTG1, CDC25C, CDK4, PLK1, PCNA, Ki67, RNASEH2B, FEN1, Cyclin D1,CD44 CDK2, CDKN3, CDC45, IQGAP3, and CDK6. Our findings indicate that using remimazolam for long term or frequently may exert potential adverse effects on patients with colon cancer under anesthesia.

## Data availability statement

The raw data supporting the conclusions of this article will be made available by the authors, without undue reservation.

## Author contributions

RW: Data curation, Formal analysis, Investigation, Methodology, Project administration, Supervision, Writing – original draft, Writing – review & editing. SL: Data curation, Formal analysis, Methodology, Writing – original draft. HH: Data curation, Methodology, Writing – original draft. QH: Data curation, Methodology, Writing – original draft. HC: Methodology, Writing – original draft. YH: Methodology, Writing – original draft. CN: Formal analysis, Writing – original draft. YR: Resources, Supervision, Writing – review & editing. HZ: Formal analysis, Funding acquisition, Writing – review & editing.
