# Supplementary material for: Transcriptomic analysis and experiments revealed that remimazolam promotes proliferation and G1/S transition in HCT8 cells
Source: Front Oncol. 2024 Apr 25;14:1345656. doi: 10.3389/fonc.2024.1345656 (PMC11079263; doi:10.3389/fonc.2024.1345656)
Supplement: Supplementary file 1 [file Table_1.docx]

| gene | Primer Sequence (5’→3’) | gene | Primer Sequence (5’→3’) |
| --- | --- | --- | --- |
| H-GAPDH-F | GGAAGCTTGTCATCAATGGAAATC | H-POLA2-F | GCTAAGCATGAACAGGTGGAGAA |
| H-GAPDH-R | TGATGACCCTTTTGGCTCCC | H-POLA2-R | AATGACGGGACAAAGACAAGGTG |
|  |  |  |  |
| H-CDC25C-F | AGCACAGCTTCTTTGTAGCACTC | H-PLK1-F | CGACTTCGTGTTCGTGGTGT |
| H-CDC25C-R | CACTGTCCACCAAGTTTCCATT | H-PLK1-R | GATGAATAACTCGGTTTCGGTG |
|  |  |  |  |
| H-FEN1-F | CTGTGCTAATGCGACACCTGAC | H-CDK1-F | AAGGGTAGACACAAAACTACAGGTC |
| H-FEN1-R | GATCTCCTCGATGCTCTTGTGC | H-CDK1-R | ATGTACTGACCAGGAGGGATAGA |
|  |  |  |  |
| H-PCNA-F | AGCCATATTGGAGATGCTGTTG | H-RNASEH2B-F | GTTCAGGAGAAGGAGCCATTTAC |
| H-PCNA-R | CTGAGTGTCACCGTTGAAGAGAG | H-RNASEH2B-R | ATCAGCCTTTATGAGGTAGTGGAG |
|  |  |  |  |
| H-KI67-F | TGGTGGGCACCTAAGACCTG | H-CDK4-F | TCTGGTGACAAGTGGTGGAACA |
| H-KI67-R | ATGGTTGAGGCTGTTCCTTGAT | H-CDK4-R | GCAGCCCAATCAGGTCAAA |
|  |  |  |  |
| H-PTTG1（1）-F | AAGACCAAGGGACCCCTCAAA | \| H-CDK6-S \| \| --- \| \|  \| | GCTGACCAGCAGTACGAATG |
| H-PTTG1（1）-R | GCATCATCTGAGGCAGGAACA | H-CDK6-A | GCACACATCAAACAACCTGACC |
|  |  |  |  |
| \| H-CDK2-S \| \| --- \| \|  \| | GTACCTCCCCTGGATGAAGAT | H-CDKN3-S | TCCGGGGCAATACAGACCAT |
| H-CDK2- A | CGAAATCCGCTTGTTAGGGTC | H-CDKN3-A | GCAGCTAATTTGTCCCGAAACTC |
|  |  |  |  |
| H-CDC45-S | TTCGTGTCCGATTTCCGCAAA | H-IQGAP3-S | GCAGCCTATGAACGCCTCA |
| H-CDC45-A | TGGAACCAGCGTATATTGCAC | H-IQGAP4-A | GGAGGGTGCAAAACAGTGG |
|  |  |  |  |

| antibody | Cat No. | antibody | Cat No. |
| --- | --- | --- | --- |
| PCNA | 10205-2-AP, proteintech | Actin | 23660-1-AP, proteintech |
| Ki67 | ab92742, abcam | tubulin | 11224-1-AP, proteintech |
| CD44 | 15675-1-AP, proteintech | CDK6 | 13331, CST |
| Cyclin D1 | 55506, CST | CDK4 | 12790, CST |
